# Ultra-Stable UiO-66 Involved Molecularly Imprinted Polymers for Specific and Sensitive Determination of Tyramine Based on Quartz Crystal Microbalance Technology

**DOI:** 10.3390/polym12020281

**Published:** 2020-01-31

**Authors:** Chi-Xuan Yao, Ning Zhao, Jing-Min Liu, Guo-Zhen Fang, Shuo Wang

**Affiliations:** 1 State Key Laboratory of Food Nutrition and Safety, Tianjin University of Science & Technology, Tianjin 300457, China; yaochixuan@mail.tust.edu.cn (C.-X.Y.); fangguozhen@tust.edu.cn (G.-Z.F.); 2 Tianjin Key Laboratory of Food Science and Health, School of Medicine, Nankai University, Tianjin 300071, China; 15891783438@163.com (N.Z.); liujingmin@nankai.edu.cn (J.-M.L.)

**Keywords:** metal–organic frameworks, UiO-66, molecularly imprinted polymers, quartz crystal microbalance, tyramine

## Abstract

A rapid method was developed to determine the content of tyramine in food on the basis of the combination of molecular imprinting technique and the metal-organic frameworks. We developed the new molecular imprinted polymers based on metal-organic frameworks UiO-66 (named UiO-66@MIPs) as the sensing recognition element, the non-molecular imprinted polymers based on UiO-66 (named UiO-66@NIPs) was synthesized according the same steps without tyramine for comparison. The characterization of obtained UiO-66@MIPs was investigated through a series of characterization experiments. The results indicated that the octahedral shaped UiO-66 was encapsulated in the sol-gel polymer film, with a desirable thermal stability and possessed a specific surface area (SSA) of 994.3 m^2^·g^−1^. The imprinting factor of the UiO-66@MIPs for tyramine was 1.956 in static experiment. This indicates the synthesized UiO-66@MIPs have outstanding performance compered to UiO-66@NIPs on the static adsorption quantity and selective adsorption affinity. It’s to make use of advantages of the synthetic materials to develop a quartz crystal microbalance (QCM) sensor for the sensitive detection of tyramine. The detection limit of the system was 61.65 μg·L^−1^ within measurable concentration range from 80 to 500 μg·L^−1^. The prepared QCM sensor was verified in selectivity and application. The UiO-66@MIPs possess good behavior on selectivity, absorptivity, and chemical stability, so the UiO-66@MIPs achieve accurate and rapid trace detection of biogenic amines in food combining with the quartz crystal microbalance.

## 1. Introduction

Biogenic amines are a class of small molecular nitrogen compounds with biological activity. The existence of a low content of biogenic amine plays an important physiological role in animals and plants [1]. Tyramine is metabolized by monoamine oxidase (MAO) [2] and is common in biogenic amines, which widely exists in fermented food or other animal-origin food matrices [3] and it has a great impact on human health [4]. Food allergens can cause a series of adverse reactions and impair human health. After excessive intake, excess tyramine contributes to an enhanced pressor effect of tyramine in human body [2]. Even, elevated blood pressure and other potential dangerous could take place following exposure to normal amounts of tyramine when using MAO inhibitors (MAOI), due to its inhibition of reducing tyramine [5]. We can see that the incidence of hypertension is closely related to the level of tyramine in the human body [6]. To take the scientific approach a step further for tyramine, it is a vital research work to measure the level of tyramine quickly and sensitively.

Some testing means, such as high-performance liquid chromatography [7] and capillary electrophoresis detection [8], required extra complicated pre-treatment processes. These testing means are not conducive to carry out spot quick detection, which are limited to the requirement of skilled technicians and sophisticated equipment. Enzyme-linked immunosorbent assay [9] (ELISA) should be determined under strict experimental condition, it will lead to inaccurate results in extreme conditions. Therefore, it is integral for the establishment of a rapid and accurate method to measure trace concentration of tyramine in food samples, reducing the risk of the control system on food safety [10].

The molecular imprinted polymers (MIPs) have the molecular recognition sites of the template molecule and, thus, obtained specific cavities for this template [11]. MIPs are workable to maximize the selectivity and sensitivity of detection sensors as recognition elements [4]. MIPs are widely used in the environmental [12], agricultural [13], food science [14], and pharmaceutical industries [15]. With the development of science and technology and the appearance of advanced instruments, the relatively homogeneous MIPs could not satisfy the growing technological requirements [16]. Recently, the researchers have turned their eyes to the molecular imprinted polymers and their hybrids based on nanomaterials due to their excellent performance in the development of sensors, hybridization MIPs, and the nanomaterial would endow some new functions to improve the performance of nanosensors [4,17,18,19,20,21,22]. Özkan et al. [4] reported a surface plasmon resonance (SPR) nanosensor incorporated MIPs and the nanocomposites of Ag@AuNPs nanoparticles/hexagonal boron nitride (HBN) nanosheets to measure the level of etoposide in urine samples, the linear range of this SPR sensor was 0.001–1.00 μg·L^−1^ and the detection limit (LOD) was 0.00025 μg·L^−1^. Kıran et al. [17] reported an electrochemical sensor to assess methyl parathion via the hybrids of MIPs and nanomaterials, this sensor was relative stable in 60 days. Atar et al. [19] reported an electrochemical sensor to determine cypermethrin based on the hybrid material of MIPs/core-shell nanoparticles/HBN nanosheets, the linearity range was from 1.0 × 10^−13^ to 1.0 × 10^−8^ mol·L^−1^ and the LOD of this sensor was 3.0 × 10^−14^ mol·L^−1^. Diethylstilbestrol (DES) is one of synthetic environmental hormones, it is quite stable and high toxicity that may be found in the industrial waste [20]. Yola et al. [21] established a molecularly imprinted sensor based on gold nanoparticles combined with two-dimensional-HBN nanosheets for DES detection. They also prepared a sensor of bilirubin measurement based on the nanocomposites of molecular imprinted polymers and functionalized carbon nitride nanotubes, this sensor was low cost and it could be used in routine analysis without disturbance [22]. Also, the molecularly imprinted technology could be coupled with quantum dots to detect a biochemical marker called cTnI of acute myocardial infarction (AMI) [23] and epinephrine [24]. Phenylethanolamine A (PEA) may cause acute toxic effects so that the researchers developed a kind of nanocomposite supported MIP materials based on Ru@Au NPs/carbon nitride nanotubes/graphene quantum dots to detect the level of PEA, the nanosensor was high selectivity and recovery even if in presence of other competitive agents in real samples. 

The quartz crystal microbalance (QCM) sensor is famous for its outstanding performance and applied in some respects of our social life [25,26,27,28], which has been used as a transducer of sensors. There was negative correlation between the binding mass of target increases and the oscillation frequency [29]. In recent years, the QCM sensor combined with molecularly imprinting polymers (MIPs) has been applied to multipurpose detection [29,30,31]. MIPs improve the selectivity of the normal QCM sensor for target molecule. Meanwhile, MIPs can concentrate the target molecules to enhance the sensitivity of the QCM sensors due to their specific cavities for target. For these reasons, the MIPs-QCM sensors have been greatly developed in the last few years.

Metal–organic frameworks (MOFs) are a class of three-dimensional porous crystalline compounds formed by self-assembly of metal ions or clusters with organic ligands. Metal–organic frameworks (MOFs) stand out for large specific surface area, tunable pore diameter, well-defined structure and high micropore volume, entrusting MOFs with huge potential in correlative technological applications, gas storage [32] and separation [33], sensing [34], catalysis [35], and drug delivery [36]. Among these porous MOFs, UiO-66 is a kind of MOF that is composed of inorganic nodes (Zr_6_-oxyhydroxide clusters) and organic linkers (1,4-benzenedicarboxylate, BDC). It was out of the ordinary due to its high constancy in common solvents [37] and high hydrothermal stability up to 500 °C [38]. The morphology and uniform sizes of UiO-66 can be adjusted by adding acid [39] and controlling the concentrations of reagents and modulators [40].

UiO-66 owned excellent chemical and thermal stability in water and can be well applied to the adsorption of various organic molecules [41]. The regulatable pore size of UiO-66 is one of its superior advantages as an adsorbent [42]. However, the adsorption range is broad-spectrum, target molecules would be not absorbed efficiently and selectively. Realizing the material functionalization of UiO-66 surface is an effective method to enhance adsorption capacity [43]. Therefore, it is necessary to improve the adsorption efficiency and specificity of tyramine on UiO-66 materials by molecular imprinting technology. The relatively high adsorption capacity of MOFs and the rapid recognition ability of imprinted materials to target molecules can be obtained by introducing high specific surface area metal–organic frameworks (MOFs) as the matrix of molecular imprinting. 

**Scheme 1:**
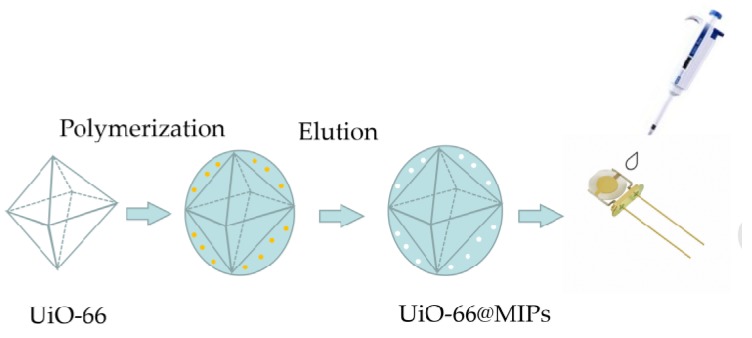
The preparation process of the nanocomposite and quartz crystal microbalance (QCM) Pt electrode.

In this work, acetic acid promoted UiO-66 was the core of the molecular imprinted polymers to for tyramine detection via a sol-gel process. Then the selective core–shell architecture polymers (UiO-66@MIPs) are used as recognition elements in the fabrication of QCM biosensor toward the target compound. The preparation process of the sensor is shown in Scheme 1. Specifically, this method combined the merits of surface molecularly imprinting technology and metal–organic frameworks. The surface-imprinted polymers also provide ideas and directions for the efficient adsorption of target compounds by quartz crystal microbalance in practical complex samples. This paves the way for the development of a new kind sensor for tyramine. The UiO-66@MIPs material was integrated within quartz crystal microbalance device to determine the trace content of tyramine for the first time.

## 2. Material and Methods

### 2.1. Instruments and Reagents

Chemicals for the experiments were from Shang Hai Aladdin Biochemical Technology Co., Ltd., Shang Hai, China: zirconium tetrachloride (98%), acetic acid (ACS, ≥99.7%), and terephthalic acid (99%) were used for UiO-66 synthesis. Tyramine (98%), 3-Aminopropyl triethoxysilane (APTES, 99%), tetraethyl orthosilicate (TEOS, 99.99% metals basis), and ammonium hydroxide (25–28% in H_2_O) were also used in the MIP synthesis. Doubly distilled water (DDW, 18.2 M·cm^−1^) was obtained from a Water Pro water purification system (Labconco, Kansas, MO, USA). Electrochemical experiments were performed with an electrochemical workstation PAR 2273 (EG&G) associated with a QCM (QCA922, Seiko EG&G) at room temperature, with a 9 MHz AT-cut quartz crystal plated with platinum electrode. UiO-66@MIP or NIP polymers were holding by Teflon that ensures that one side of the Pt electrode can only connect to the measured liquid while the other side is insulated.

### 2.2. Synthesis of UiO-66 and UiO-66@MIPs/NIPs

UiO-66 was obtained by solvothermal synthesis method [44]. The nanoparticles of molecular imprinted polymers based on UiO-66 (UiO-66@MIPs) were prepared with UiO-66 through sol-gel molecularly imprinted polymerization. The artificial materials were made of the functional monomer of APTES and the cross-linker of TEOS toward tyramine (“template”) as schematically. Tyramine (100.0 mg) was firstly put into 5.0 mL ethanol with fully dissolution. APTES (0.687 mL) was added into the reaction system under continuous mixing. The prepolymer formed after stirring 30 min, 200.0 mg of UiO-66 and 0.664 mL of TEOS were added into above mixture to continue the mixing process. Finally, 0.4 mL of 3.0 mol·L^−1^ aqua ammonia participated in reaction to catalyze the formation of highly crosslinked products. It took 24 h for polymerization process and the polymers were aged for 10 h under vacuum drying. The obtained polymers were eluted ethanol and water (1:1) until tyramine would be not detected by ultraviolet spectrophotometer, then dried under vacuum overnight. The nanoparticles of non-molecular imprinted polymers based on UiO-66 (UiO-66@NIPs) were synthesized following the same step as UiO-66@MIPs, only without adding template molecules.

### 2.3. Adsorption of Tyramine.

The adsorption method was conducted as follows. For static experiment, UiO-66@MIPs or UiO-66@NIPs (4.0 mg) was mixed with 2.0 mL buffer (phosphate buffered saline, pH = 7.4) containing different concentrations of tyramine (5.0–140 mg·L^−1^) in microcentrifuge tubes. The mixture was shaken for 12 h and centrifuged twice at 12,000 rpm for 15 min. For adsorption kinetic experiment, 4 mg MIPs or NIPs was accurately weighed and placed in 2.0 mL 100 mg·L^−1^ tyramine PBS standard solution, shaking for 10 min to 12 h at room temperature. The remaining tyramine in each supernatant fluid was estimated by the ultraviolet spectrophotometer at 225 nm to examine. The adsorption property of UiO-66@MIPs (*Q*, mg·g^−1^) was calculated by Equation 1:

*Q* = (*C_i_* − *K* × *C_f_*) × *V/W* (1)

where *C_i_* is the original concentration of tyramine in PBS solution (mg·L^−1^), *C_f_* is the final concentration of tyramine after adsorption (mg·L^−1^), *K* is dilution multiple in detection, *W* is the amount of polymers (mg), and *V* represented the volume of adsorbed solution (L). 

### 2.4. QCM Measurements

Before being modified, quartz crystal gold electrode should be cleaned in ethanol by ultrasound, and let it soak in a new “piranha” solution (30% H_2_O_2_/concentrated H_2_SO_4_ = 1:3, *V/V*) for 5 min. After washing thoroughly, the quartz crystal Pt electrode was dried with nitrogen and assembled into the PTFE fixator. 10 mg of UiO-66@MIPs or UiO-66@NIPs was accurately weighed and mixed into 2.0 mL dichloromethane (containing 2.0 mg polyvinyl chloride) in centrifugal tubes, respectively. The mixture was uniformly distributed by vortex oscillation and ultrasound until a uniform suspension was obtained. The mixture (5.0 μL) would be uniformly coated on Pt electrode layer. The modified electrode layer was dried and kept at room temperature, fixed in quartz crystal microbalance prior to use.

The assembled sensor was immersed in PBS buffer (pH = 7.4, 25 mL) under magnetically stirring at 200 rpm until the initial value of resonant frequency (*f_0_*) was obtained after reaching equilibrium. Minim tyramine solution was transferred to the cell, a new stable frequency change (*f*) was obtained after adsorption equilibrium of target by QCM sensor. After each detection process, the modified Pt electrode was eluted by 1.0 mol·L^−1^ HCl and deionized water up to the frequency change become stable again. We use this reusable modified Pt electrode to carry out the successive determination and every single experiment was repeated at least three times.

### 2.5. Sample Preparation

Standard addition technique was utilized for the determination of tyramine in terms of validation. Soy sauce was chosen as actual sample to evaluate the practical application value of UiO-66@MIPs-QCM sensor for tyramine measurement. Soy sauce was purchased from the native market and added tyramine standard solution of 200 μg·L^−1^, 300 μg·L^−1^ and 400 μg·L^−1^, separately. The added samples were extracted and degreased to remove unnecessary fat and protein. Through 0.22 μm filtration, the prepared samples were detected by the prepared QCM sensors. *Δf* was taken notes for analysis until the change of frequency was less than 1 (*Δf* = *f_0_* − *f*). 

## 3. Results and Discussion

### 3.1. Characterization

MOFs were provided with permanent porosity after removal of guest molecules by activation. Moreover, MOFs have the high porosity of the material skeleton, low density and large specific surface area [45]. UiO-66 has productive chemical stability [46], therefore its crystalline structure can be maintained under strong acid and alkaline corrosion environment. Based on the above-mentioned advantages, we choose UiO-66 as the supporter of molecularly imprinted polymers.

**Figure 1:**
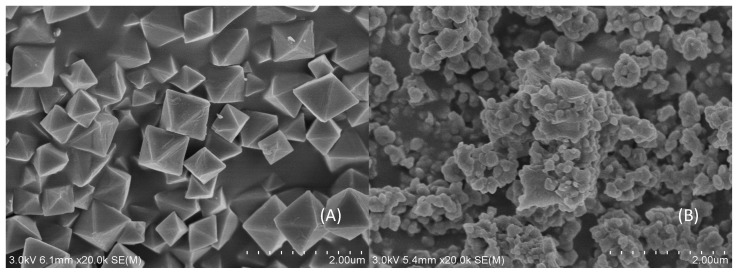
SEM images of synthesis samples: (**A**) UiO-66 and (**B**) UiO-66@MIPs (molecular imprinted polymers).

Acetic acid promoted UiO-66 presented more regular octahedron crystalline shape than the synthetic samples without acid regulator [39], and the particle was uniform size with good dispersion performance and no agglomeration, which the average particles size is about 734 nm (Figure 1A). After polymerization, it is obvious that the clear octahedral structure of UiO-66 had disappeared in Figure 1B. There is a film wrapped around the surface of UiO-66 crystalloid, the aggregation actually occurs between UiO-66 crystals. The imprinted layer was wrapped on the outer of UiO-66 crystals, the size of particles becomes larger—about 757 nm. The obtained larger nanoparticles indicated that the MIPs layer was successfully formed outside UiO-66.

**Figure 2:**
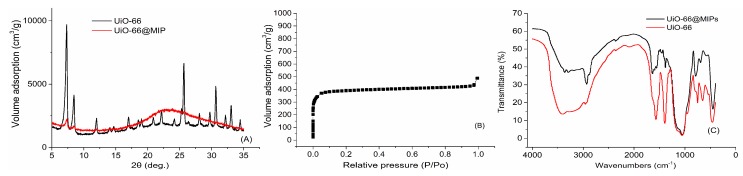
The Characterization of the materials: (**A**) the XRD patterns of UiO-66 and UiO-66@MIPs; (**B**) the N_2_ patterns of UiO-66; and (**C**) the FTIR patterns of UiO-66 and UiO-66@MIPs.

Figure 2A is the XRD spectra of MOFs and UiO-66@MIPs. The XRD patterns of UiO-66 show the strong and narrow typical UiO-66 characteristic peaks, which at 2θ = 7.35° and 8.50°, indicating well-quality crystal structures. The patterns of UiO-66 were in good agreement with the previous literature report [47]. For UiO-66@MIPs, we can observe clearly after surface-imprinting process, the XRD peaks intensity of UiO-66 almost decreased, forming the coating polymerization successfully.

The size and crystallinity of UiO-66 can be adjusted by changing the proportion of acetic acid during the synthesis process. The N_2_ adsorption graph (Figure 2B) shows the isothermal adsorption curve of UiO-66 we prepared. It can be seen that the synthesized UiO-66 is a typical type I adsorption isotherm, which indicates the acetic acid promoted UiO-66 belongs to microporous material. At the same time, the specific surface area of UiO-66@MIPs was 19.258 m^2^·g^−1^, much lower than the unmodified UiO-66 (994.3 m^2^·g^−1^). These characterizations contributed to the successfully prepared UiO-66. The results demonstrate the existence of a surface-imprinted layer. 

Fourier transform infrared spectroscopy (FTIR) is a powerful method to characterize the properties of organic groups. UiO-66 and UiO-66@MIPs were characterized by FTIR (Figure 2C). The pattern of UiO-66 shows the carbon–carbon double bond and carboxyl group structure of the phenyl ring skeleton from terephthalic acid. Compared with UiO-66, MIPs have weak C=C vibrations at 1620 cm^−1^, intensity of the strong absorption peaks weakened at 3400 cm^−1^ and 1570 cm^−1^, suggesting that polymerization and hydrolysis reaction happened on the surface of UiO-66 and the existence of molecularly imprinted polymers.

### 3.2. Adsorption of Tyramine-MIPs

**Figure 3:**
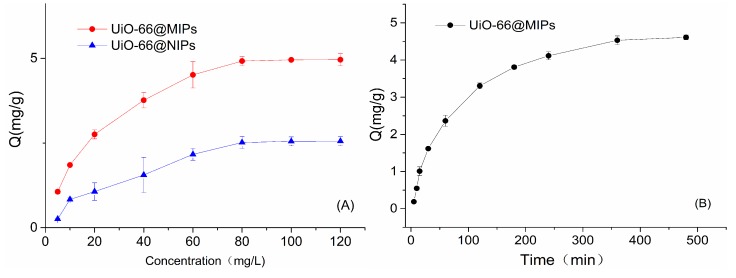
Binding isotherm of UiO-66@MIPs and UiO-66@NIPs for tyramine: (**A**) static adsorption curve and (**B**) dynamic adsorption curve.

As shown in Figure 3A, the adsorption capacities of the prepared materials (UiO-66@MIPs and UiO-66@NIPs) increased with tyramine concentration. In the initial stage, the equilibrium adsorption capacities increased rapidly, the adsorption capacity of MIPs to tyramine was always higher than that of NIPs. Then the adsorption capacities increased slowly until they reached saturation. UiO-66@MIPs have better adsorption capacity for tyramine than UiO-66@NIPs. The equilibrium adsorption capacity of UiO-66@MIPs for tyramine (*Q_MIPs_* is 4.92 mg·g^−1^) is 1.95 times as much as UiO-66@NIPs (*Q_NIPs_* is 2.51 mg·g^−1^) at low level (80 mg·L^−1^). UiO-66@MIPs have better adsorption capacity for tyramine than non-imprinted polymers. This may be due to better matching of recognition sites and templates on imprinted surfaces, allowing the imprinted layer to absorb more template molecules. On the other hand, based on the surface imprinting technology, the thinner the thickness of imprinted shell is, the faster the mass transfer speed UiO-66@MIPs it has, while UiO-66@NIPs are mainly non-specific adsorption.

Furthermore, the dynamic adsorption kinetic of UIO-66@MIPs was also studied (Figure 3B). The adsorption time of UIO-66@MIPs was investigated under the same concentration. The result shows that the adsorption capacity reached 1.78 mg·g^−1^ at 30 min, basically reaching equilibrium state in 4 h. The adsorption rate of the prepared molecularly imprinted polymers was relatively fast, mainly because the mass transfer rate of UiO-66@MIPs was high due to the comparative soft structure of the sol-gel molecularly imprinted polymer, the template molecules are more easily adsorbed.

### 3.3. The Performance of QCM Sensor

**Figure 4:**
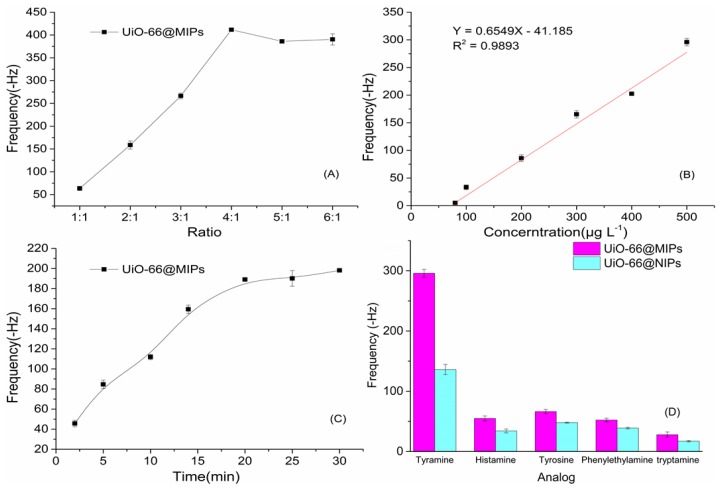
The performance of UiO-66@MIPs QCM sensor: (**A**) Effects of different ratios of PVC to MIPs on QCM sensors; (**B**) Frequency changes of UiO-66@MIPs QCM sensor in tyramine standard solutions; (**C**) Response time of the QCM sensor; and (**D**) The selective performance of UiO-66@MIPs/UiO-66@NIPs QCM sensors.

UiO-66@MIPs and polyvinyl chloride (PVC) are the main components of modified membranes on the surface of the gold electrode. Under a certain amount of coating (10.0 μL), different amounts of MIPs added would affect the imprinted binding sites of the modified membranes. Therefore, the amount of imprinted polymer should be maximized while the sensitive film is closely modified on the quartz wafer. In 500 mg·L^−1^ tyramine standard solution, the frequency changes of the sensors were measured under different coating proportions of PVC and polymers. From Figure 4A, the frequency was up to maximum variation when UiO-66@MIPs was four times the quantity of PVC. Meanwhile, the frequency of the sensor based on UiO-66@MIPs became stable gradually in PBS buffer, target solution was dropped into the buffer and the frequency of the sensor decreased. Also, we can find that the sensor handily took up tyramine in 10 min (Figure 4C), reached the constant value after 16 min.

Quartz crystal microbalance sensors based on UiO-66@MIPs was used to evaluate the binding capacity of tyramine at different concentrations. As shown in the Figure 4B, *Δf* value increased with increasing concentrations of tyramine. This could be attributed to the binding sites of UiO-66@MIPs. The generated sites could fix the target molecule specifically ascribed to porous structure and effective chemical groups on MIPs film. The frequency change of UiO-66@MIPs sensor response is linear with the concentration of tyramine in range of 80–500 μg·L^−1^ under certain condition. In addition, the LOD was 61.65 μg·L^−1^.

The specificity is a major challenge for rapid detection methods to ensure the accuracy. The potential competitive agents were unavoidable as the coexisting substances during the analysis. Tyramine, histamine, and tryptamine are the products after tyrosine decarboxylation [48]. Histamine, tryptamine, β-phenylethylamine, and tyramine all belong to monoamines and were common in food samples [49,50,51,52]. Thus, we chose histamine, tryptamine, β-phenylethylamine, and tyrosine to verify selectivity of the sensors. Under the same concentration, we can see the UiO-66@MIPs sensor has the highest response to tyramine from Figure 4D, but also has weak response to other compounds. Some functional groups similar to that of tyramine can interact with the binding sites in the cavities of polymer particles on the modified membranes. However, due to the structural differences between them, these competitive molecules could not fully enter the imprinted cavities and combine the active binding site in the cavities, which results in the response of every analog is weaker than that response of tyramine. For UiO-66@NIPs sensor, either tyramine or other compounds, the responses were very small due to the no suitable sites for tyramine. Further, we defined the imprinting factor (IF) for selectivity estimate in QCM experiment,

*IF_QCM_* = *Δf_MIP_/Δf_NIP_* (2)

where *Δf*_MIP_ and *Δf_N_*_IP_ are respective response frequency variations of the five analytes. The detection results that the impact factor of tyramine was 2.17, obviously higher than the result of any other analog (the impact factors of histamine, tyrosine, tryptamine, β-phenylethylamine, and tryptamine were 1.60, 1.38, 1.34, and 1.81, separately), indicating that the UiO-66@MIPs-QCM sensor had excellent specificity for tyramine. This showed the UiO-66@MIPs-QCM sensor was of excellent selectivity by frequency change, and also proves the successful application of molecularly imprinted technology and QCM technology.

### 3.4. Evaluation of sample analysis and comparison of the methods

After sample pretreatment, the content of tyramine in soy sauce samples, which were added at three concentrations, was tested by UiO-66@MIPs–QCM sensor. The recovery for the detection results ranged from 86.74% to 93.62% in Table 1, expressing that as-prepared UiO-66@MIPs-QCM sensor could be used in practical application for accurate tyramine determination. The sensor performance was compared with previous reported results in Table 2. Compared with these studies, the established method was higher sensitivity or wider detection range by improved specificity of MOFs and enhanced adsorption property of the molecularly imprinted polymers.

## 4. Conclusions

In this paper, a QCM sensor based on UiO-66@MIPs were constructed and applied for tyramine detection. The material was characterized and the detection conditions were optimized, and it was applied to monitor the content of tyramine in soy sauce samples. The results showed that the sensor has the best response when the mass ratio of membrane material was 4:1 and the adsorption time was 16 min. The linear response of the sensor was detected in tyramine standard solution of 80–500 μg·L^−1^, the regression equation was obtained: y = 0.6549x − 41.185 (*R*^2^ = 0.9893), and its LOD was 61.65 μg·L^−1^. The UiO-66@MIPs QCM sensor had high specificity for tyramine. At last, tyramine was detected five times in succession in soy sauce samples, and the calculated recoveries were in the range of 88.82–95.53%. In this work, the feasibility between MIT and QCM technology were proved through preliminary research, so did the practicability of MOFs–MIPs. It is believable that the combination between MOFs and molecular imprinted technology is favorable for highly sensitive sensor system fabrication to tyramine.

## Figures and Tables

**Table 1:** Cycling performance of the sensor tested in the samples (*n* = 3).

Dosage (μg·L^−1^)	Measured Quantity (μg·L^−1^)	Rate of Recovery (%)	RSD (%)
200	182.96	91.47	3.7
300	280.88	93.62	2.2
400	346.95	86.74	1.1

**Table 2:** Comparison of different analytical approaches for tyramine determination.

Method	Linear Range (μg·L^−1^)	LOD (μg·L^−1^)	Reference
HPLC	0.05–0.25	0.06	[53]
DPV	(0.685–24.69) × 10^3^	85.05	[54]
Amperometry	(1.37–68.0) × 10^3^	274.36	[55]
Colorimetry	5–40	2	[56]
QCM	80–500	61.65	This work
